# Hereditary Spastic Paraplegia and Intellectual Disability: Clinicogenetic Lessons From a Family Suggesting a Dual Genetics Diagnosis

**DOI:** 10.3389/fneur.2020.00041

**Published:** 2020-02-14

**Authors:** Sergio Aguilera-Albesa, Ana Belén de la Hoz, Nekane Ibarluzea, Andrés R. Ordóñez-Castillo, Olivia Busto-Crespo, Olatz Villate, María Asunción Ibiricu-Yanguas, María E. Yoldi-Petri, Iñaki García de Gurtubay, Guiomar Perez de Nanclares, Arrate Pereda, María Isabel Tejada

**Affiliations:** ^1^Paediatric Neurology Unit, Department of Paediatrics, Navarra Health Service Hospital, Pamplona, Spain; ^2^Navarrabiomed Health Research Institute, Pamplona, Spain; ^3^Biocruces Bizkaia Health Research Institute, Barakaldo, Spain; ^4^Clinical Group Affiliated With the Centre for Biomedical Research on Rare Diseases (CIBERER), Valencia, Spain; ^5^Department of Neurology, Navarra Health Service Hospital, Pamplona, Spain; ^6^Department of Physical Medicine and Rehabilitation, Navarra Health Service, Pamplona, Spain; ^7^Molecular Genetics Laboratory, Genetics Service, Cruces University Hospital, Osakidetza Basque Health Service, Barakaldo, Spain; ^8^Department of Neurophysiology, Navarra Health Service Hospital, Pamplona, Spain; ^9^Rare Diseases Research Group, Molecular (Epi)Genetics Laboratory, Bioaraba Health Research Institute, Araba University Hospital, Vitoria-Gasteiz, Spain

**Keywords:** intellectual disability, hereditary spastic paraplegia, SPG 31, copy number variants, dual genetic etiology

## Abstract

Hereditary spastic paraplegias (HSPs) are a heterogeneous group of genetic disorders with spastic paraparesis as the main clinical feature. Complex forms may co-occur with other motor, sensory, and cognitive impairment. A growing number of loci and genes are being identified, but still more than 50% of the patients remain without molecular diagnosis. We present a Spanish family with autosomal dominant HSP and intellectual disability (ID) in which we found a possible dual genetic diagnosis with incomplete penetrance and variable expressivity in the parents and three siblings: a heterozygous duplication of 15q11.2–q13.1 found by array CGH and a novel missense heterozygous change in *REEP1* [c.73A>G; p.(Lys25Glu)] found by whole exome sequencing (WES). Following the standard genetic diagnosis approach in ID, array CGH analysis was first performed in both brothers affected by spastic paraparesis and ID from school age, and a heterozygous duplication of 15q11.2–q13.1 was found. Subsequently, the duplication was also found in the healthy mother and in the sister, who presented attention deficit/hyperactivity disorder (ADHD) symptoms from school age and pes cavus with mild pyramidal signs at 22 years of age. Methylation analysis revealed that the three siblings carried the duplication unmethylated in the maternal allele, whereas their mother harbored it methylated in her paternal allele. Functional studies revealed an overexpression of *UBE3A* and *ATP10A* in the three siblings, and the slightest cognitive phenotype of the sister seems to be related to a lower expression of *ATP10A*. Later, searching for the cause of HSP, WES was performed revealing the missense heterozygous variant in *REEP1* in all three siblings and the father, who presented subtle pyramidal signs in the lower limbs as well as the sister. Our findings reinforce the association of maternally derived *UBE3A* overexpression with neurodevelopmental disorders and support that a spectrum of clinical severity is present within families. They also reveal that a dual genetic diagnosis is possible in patients with presumed complex forms of HSP and cognitive impairment.

## Background

Hereditary spastic paraplegias (HSPs) are a heterogeneous group of genetically determined disorders with progressive spastic paraparesis as the main clinical feature (1, 2). From a clinical point of view, an isolated upper motor neuron syndrome defines *pure* forms of HSPs, while *complex* forms may associate other motor, sensory, and cognitive involvement (3, 4). Population-based studies showed that complex forms of HSPs represent more than 50% of all HSP cases, with ataxia and sensory involvement as the most common signs. Cognitive impairment is present only in 8% of all cases, but it is related to a more severe disease (5), overlapping with other neurodegenerative disorders that present with spastic paraparesis and cognitive deficits (6). This clinical diversity reflects the underlying genetic background in HSPs, with more than 70 loci and 50 genes that can be inherited through autosomal dominant and recessive, X-linked, and maternal modes of transmission (7). However, genetic causes remain obscure in more than 50% of the cases (6).

On the other hand, copy number variants (CNVs) in the human genome were proven to be at the origin of many neurodevelopmental disorders, including intellectual disability (ID) (8), and among them, the chromosome region 15q11–13 is prone to duplications and deletions. This region comprises a cluster of imprinted genes that are crucial for the normal neurodevelopment (9). The phenotype associated with the 15q11–13 duplication is characterized by developmental delay, autism, seizures, learning difficulties, and motor skill impairments, among others, and show a marked clinical variability (10).

In this growing landscape of clinical and genetic heterogeneity in HSPs and ID, we contribute with an exceptional family whose siblings are affected by HSP and ID with a variable expressivity, outstanding a dual genetic etiology: an unmethylated 15q11.2–q13.1 duplication inherited from the mother and a *REEP1* variant inherited from the father.

## Case Presentation

**Figure 1:**
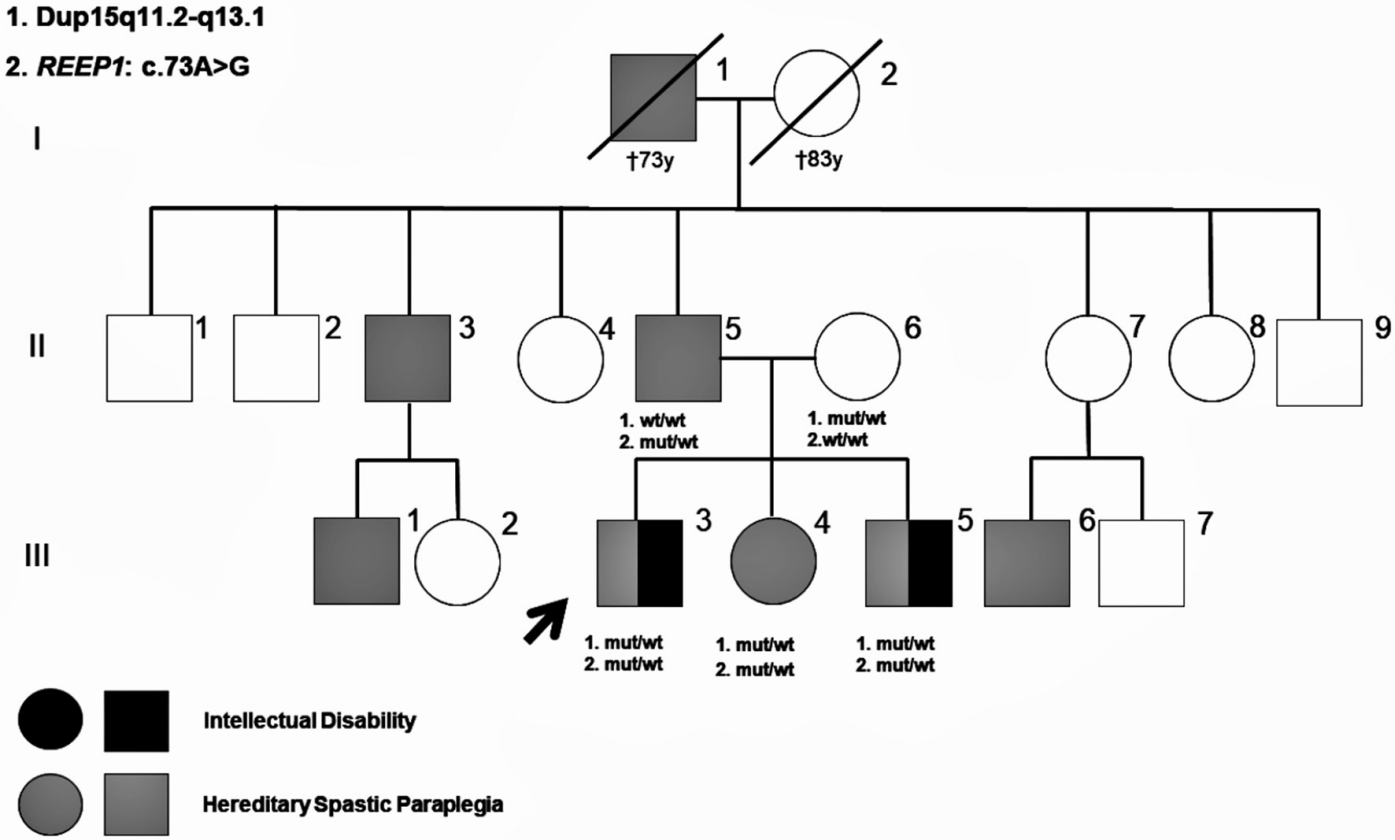
Pedigree of the present family. Molecular genetic results of the studied family members are shown in the pedigree. Patients reported as having signs of hereditary spastic paraplegia (HSP) are also shown.

We describe a non-consanguineous family with three siblings, two of them males with intellectual disability and spastic paraparesis and one female with behavioral problems and pes cavus (Figure 1). The molecular study of the two males began in 2016 in the context of a research project (PI14/00321) approved by the ethics committee for clinical research of Euskadi-Basque Country (CEIC-E: PI2014192). Informed consent was obtained from all of the patients and relatives participating in the study, before the extraction of peripheral blood samples for genetic analysis, and they provided written consent to publish the report.

### Patients

Patient III-3 (Figure 1) is a 25-year-old male without history of pregnancy or delivery complications. The development was considered within normal limits until 4 years of age, when parents and teachers reported learning difficulties and behavioral problems, characterized by poor socializing skills, and hyperactivity. At that time, physical examination was unremarkable, except for mild clumsiness. Brain CT, karyotype, and subtelomeric region tests were normal, but electroencephalogram (EEG) showed generalized paroxysmal activity with no seizures recorded or reported. Starting from 8 years, he presented with frequent falls, progressive abnormal gait, and pyramidal signs in the lower limbs. Transcranial magnetic stimulation (TMS) revealed abnormal central motor conduction to both legs. Brain and spine MRI, nerve conduction studies (NCS), electromyography (EMG), and standard metabolic studies, including urine guanidinoacetate, were normal. At 14 years old, he started with tonic–clonic and atonic seizures, associated with bifrontal spike and wave's discharges on EEG, with normal background activity and no electroencephalographic criteria for Lennox–Gastaut syndrome. Seizures were refractory to anti-epileptic drugs in the following years. At 25 years, he presented a slowly progressive spastic paraparesis with autonomous deambulation and intellectual disability (IQ: 55), without speech impairment or autism traits. Epilepsy is partially controlled with valproic acid, phenobarbital, and lamotrigine. Somatosensory evoked potentials (SSEP) revealed abnormal central conduction velocities to both legs. A recent NCS showed mild reduced motor and sensory nerve conduction velocities in several lower limb nerves, indicating polyneuropathy.

Patient III-5 (Figure 1) is a 20-year-old male, without history of pregnancy or delivery complications and apparently normal development in the first 3 years of life. Starting from 5 years old, parents and teachers reported learning difficulties and a clumsy gait. At that time, physical examination showed pyramidal signs in the lower limbs suggesting mild spastic paraparesis. TMS revealed abnormal central motor conduction to both legs. Brain and spine MRI, EEG, NCS, EMG, karyotype, and subtelomeric regions tests were normal. The cardiological exam showed mild interventricular communication without clinical symptoms. Starting from 8 years of age, he needed academic support in regular school. A multilevel orthopedic surgery was performed in the lower limbs at 17 years old, with improvement of the autonomous gait. At the age of 20, he presented a slowly progressive spastic paraparesis that needs walking support for long distances and mild intellectual disability (IQ: 67) with neither speech impairment nor autism features. No seizures were reported. SSEP revealed abnormal central conduction velocities to both legs. Recent NCS revealed a slight reduced sensory nerve conduction velocity in both sural nerves.

Patient III-4 (Figure 1) is a 22-year-old female, with normal pregnancy, delivery, and neurodevelopment during the first years of life. She accepted to be evaluated for genetic counseling following familiar molecular studies. She presented with behavioral problems from school age, characterized by attention deficit, impulsivity, and hyperactivity. An ADHD (attention deficit/hyperactivity disorder) diagnosis was made starting from 8 years old, with normal IQ. From teenage she referred social anxiety symptoms, clumsiness, and feet pain with long walks. At the age of 22, physical examination revealed pes cavus with brisk tendon reflexes and mild pyramidal signs, without muscle wasting, spasticity, or low vibration sense. NCS, SSEP, and TMS did not show abnormalities.

The father (II-5 in Figure 1) is a 55-year-old male, without any relevant history. He referred feet pain with long walking distances 2 years ago. Physical examination revealed subtle pyramidal signs in the lower limbs, with brisk tendon reflexes, Rossolimo sign but flexor plantar response and normal vibration sense. He has mild pes cavus with normal NCS, SSEP, and TMS. The mother (II-6 in Figure 1) is a healthy 50-year-old female without any learning difficulties at school age as she reported.

On further exploring the family history, a brother (II-3) and a nephew (III-1) of the father were reported to be suffering from chronic gait problems that may suggest spastic paraparesis, but they refused to be examined. Another nephew (III-6) was diagnosed with spastic paraparesis, but the molecular study was not performed either. In relation to information about maternal members, the mother reported that none of their relatives was diagnosed of a neurodegenerative disorder.

### Molecular Methods

DNA from all the analyzed patients were extracted using standard procedures and quantified by means of Invitrogene Qubit fluorometer.

### Array CGH

DNA samples were tested against normal DNA (Agilent Reference DNA) by Comparative Genomic Hybridization array (CGH Array) using qChip Post® Postnatal Research Microarray 8×60K, which was designed and optimized by qGenomics-Genomics for Human Health Laboratories (Barcelona, Spain).

Both labeling and hybridization were performed following the standard operating procedures (SOPs). The process was subject to internal quality control. The Feature Extraction software version 11.0.1.1 was used to extract and normalize data from microarray image files (TIFF file) of scanned Agilent CGH microarrays. Quality control metrics were within the normal ranges. Data were processed using the Cytogenomics software v4.0 (Agilent Technologies), with the statistical algorithm ADM-2, sensitivity threshold 5, and at least three consecutive aberrant probes.

### CNV Validation by Quantitative Real-Time PCR (ReTi-qPCR)

ReTi-qPCR was used for gene quantification by the relative method 2^−ΔΔCt^. For this purpose, *RPP30* was used as the reference gene sequence because it is located in a highly conserved region of chromosome 10. Two genes were analyzed by this method: *NIPA1* and *MAGEL2*. Each RT-qPCR was performed in a final volume of 20 μl using Brilliant II SYBR® Green QPCR Master Mix (Agilent Technologies) and the 7900HT Fast Real-Time PCR System, in accordance with the manufacturer's instructions. Exon–exon junction primers were designed using the Primer 3 Plus software.

### Methylation-Specific Multiplex Ligation-Dependent Probe Amplification (MS-MLPA)

Allelic dosage and methylation analyses of PWS/AS critical region of chromosome 15q11 were carried out by MS-MLPA using SALSA ME028-C1 kit (MRC-Holland, Amsterdam, The Netherlands). The analysis of MS-MLPA PCR products was performed on ABI3500 genetic analyzer and analyzed using the GeneMapper v.4.1. The analysis of raw data was carried out using an Excel-based in-house program based on the manufacturer's recommendations.

### Expression Studies in mRNA

PAXgene Blood RNA Kit (PreAnalytix) was used to isolate and purify intracellular RNA from blood stabilized in PAXgene Blood RNA tubes, following the manual procedure. cDNA was obtained from RNA with SuperScriptTM II Reverse Transcriptase (Invitrogene) and quantified by Qubit. Each reaction was performed in a total volume of 20 μl in the 7900HT Fast Real-Time PCR System. *GADPH* was used as housekeeping gene, and the studied genes were *NIPA1, SNRPN, UBE3A*, and *ATP10A*. All genes were analyzed with a reference DNA. A relative quantification was carried out (2^−ΔΔCt^).

### Exome Sequencing and Data Analysis

Whole exome sequencing was performed with the strategy of family trio (father, mother, and the older brother or Index Case III-3), using the Ion Proton sequencer (Thermo Fisher Scientific, P/N 4476610, Waltham, MA, USA). The Torrent Suite software (version 5.12.0) was used to align reads to the UCSC hg19 reference sequence. After uploading BAM files to the Ion Reporter (version 5.10.5.0) and launching the “AmpliSeq Exome trio” protocol, all variants were identified. Variants were filtered for genes associated with ID/HSP.

### Validation of SNV by Sanger Sequencing

All variants selected were confirmed by Sanger sequencing in parents and siblings.

## Results

### 15q Duplication

**Figure 2:**
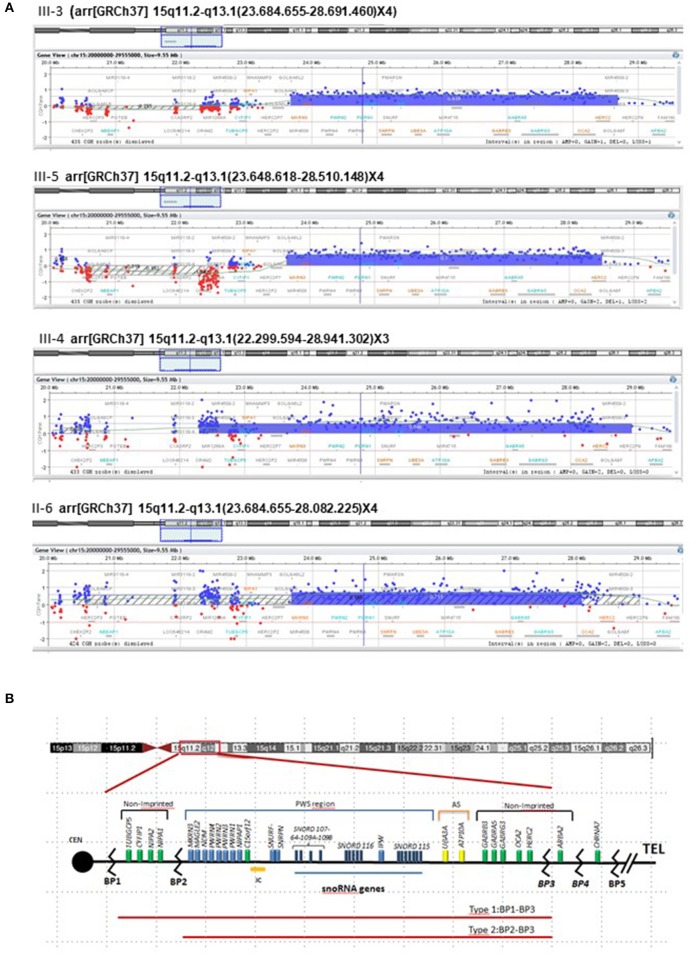
Analysis of array CGH. **(A)** Array CGH disclosed microduplication in chromosome 15 of the three siblings (III-3, III-4, and III-5) and their mother (II-6). Array CGH profile of chromosome 15 showed a duplication of ~5 Mb at 15q11.2–q13.1. The X axis (top) = represents clonal distance (in Mb) from the centromere. Genomic position of the aberration for GRCh37/hg19 reference genome is described in detail for each case in their formula above each chromosome 15 image. **(B)** The critical region for PWS on chromosome 15, with the main genes indicated in blue, expressed from paternal chromosome (blue bar). Black lines between BP1 and BP2 and from the *GABRB3* gene to B4 indicate non-imprinted genes, and the yellow bar is the maternally imprinted region (*UBE3A* and *ATP10A*—shown in yellow). IPW, an RNA transcript lies within the snoRNA region, does not encode a protein, but is paternally expressed only. BP, breakpoint; Cen, centromere; tel, telomere.

The array CGH analysis performed in III-3 revealed a duplication in the chromosomal region 15q11.2–q13.1 (OMIM number 608636), and consequently, this aberration was further studied in the brother, sister, and mother as well. The extension of the duplication showed a certain degree of size variability, being 4.86 Mb as the minimal size of the duplication in III-5 (base position chr15: 23.648.618–28.510.148) and the maximum size of 6.64Mb in his sister (base position chr15: 22.299.594–28.941.302) (Figure 2A), although the log2 (ratio) values of the probes in the upstream region of this patient were not consistent. The break-points are in accordance with those reported as “Type II” by Roberts et al. (11) going from the breakpoints BP2 to BP3 (Figure 2B) and including the Prader–Willi/Angelman critical region (PWACR) and a cluster of imprinting genes involved in those syndromes (*UBE3A* and *ATP10A, GABRB3, GABRA5*, and *GABRG3*). Among those genes, there are two maternally expressed genes, *ATP10A* and *UBE3A*.

The duplication was confirmed by ReTi-qPCR in DNA, checking *NIPA1* and *MAGEL2*. In all cases (mother, two sons, and daughter), the duplication did not include *NIPA1* demonstrating that the upstream end of the duplication in the sister was not real.

### Methylation Patterns

Allele dosage analysis by MS-MLPA confirmed the presence of a heterozygous duplication of at least 4.6 Mb in the three siblings and mother (Supplementary Figure 1) showing the absence of *NIPA1* inside the duplication in all the individuals studied, including the sister. According to the methylation pattern, the three siblings carried the duplication in the maternal allele, whereas their mother harbored it in her paternal allele (Supplementary Figure 1). Therefore, the duplication probably appeared from an anomalous recombination at BP2 and BP3 on the mother's paternal allele.

### Expression Studies

**Figure 3:**
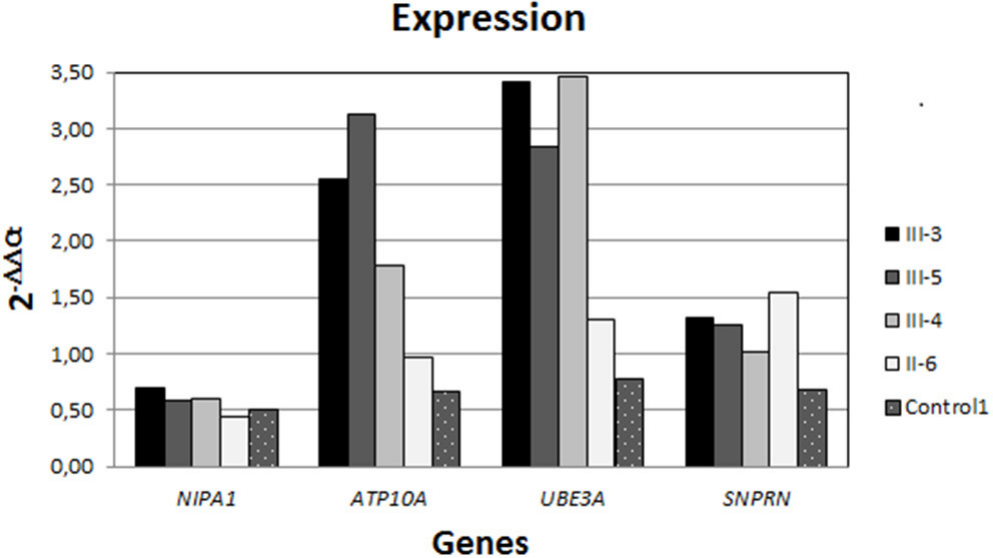
Expression analysis by RT-qPCR. All individuals showed a similar level of *NIPA1* expression (non-methylated); nevertheless, the expression of both *ATP10A* and *UBE3A* (maternally expressed) is strongly higher in the siblings than in the mother, who had similar levels to the controls. In contrast, *SNPRN* (paternally expressed gene) was rising in the mother with respect to the offspring and controls.

Expression analysis by RT-qPCR showed a similar level of expression for *NIPA1* (gene expressed in both chromosomes) in the mother and her offspring, while significant differences were found in *SNRPN, UBE3A*, and *ATP10A* (Figure 3). The *SNRPN* gene is transcribed exclusively from the paternally inherited chromosome and showed higher expression in the mother than in her offspring. In contrast, *UBE3A* and *ATP10A* (both maternally expressed genes) were overexpressed in the three siblings, whereas in the mother, the expression level of those genes were similar to the controls. Regarding *ATP10A*, the two brothers have a higher expression than the sister does, whereas for *UBE3A*, the expression is higher in the three offspring with respect to the mother or controls.

### Whole Exome Sequencing and Sanger Sequencing

**Figure 4:**
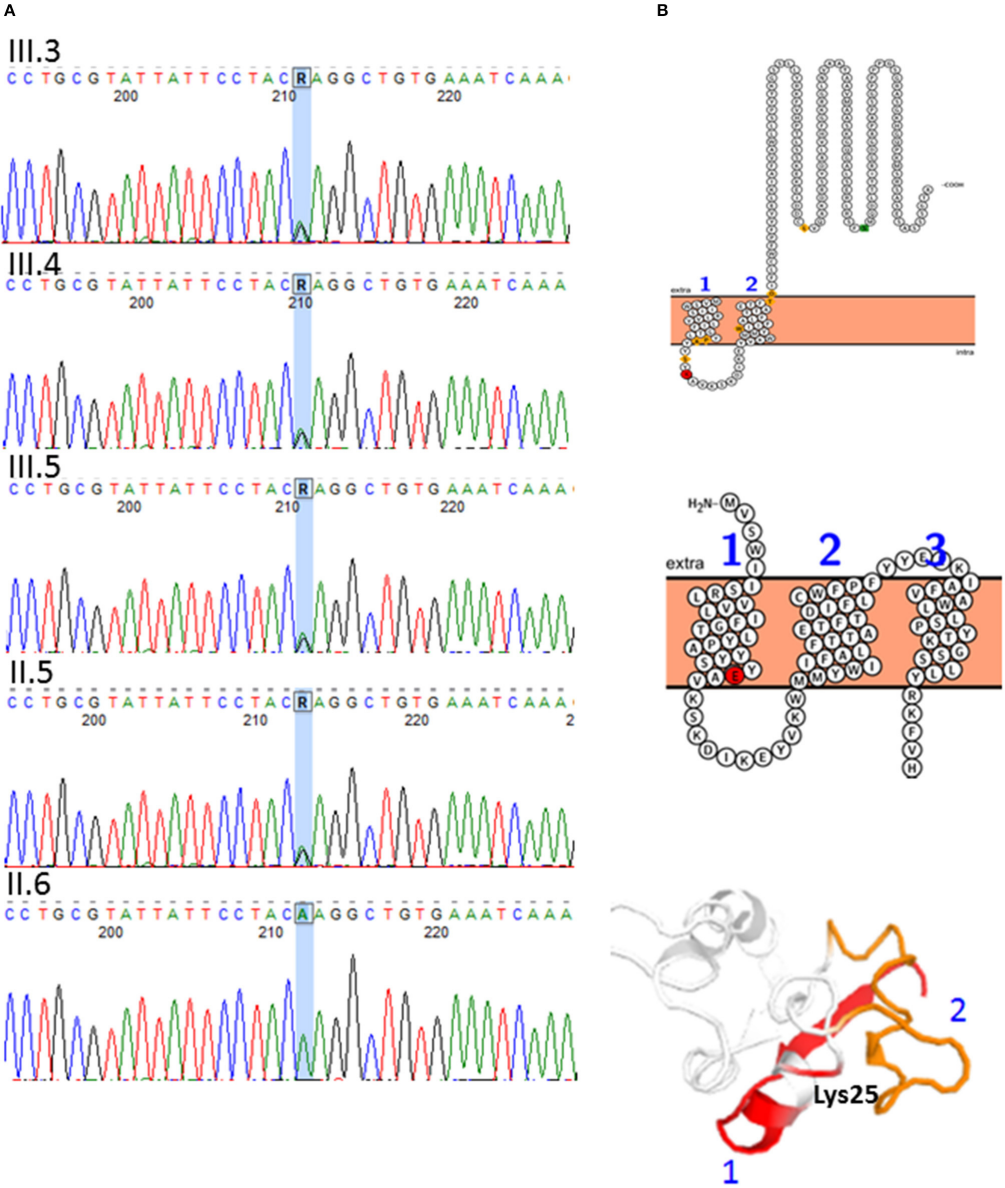
A novel variant was found in the *REEP1* gene: c.73A>G; p.(Lys25Glu) (NM_022912.2), chromosomal location 2p11.2. **(A)** DNA sequence electropherograms showing *REEP1* c.73A>G variant. The variant is present in the father (II-5) and siblings (III-3, III-4, and III-5) **(B)** REEP1 *in silico* protein modeling. Conformational change caused by p.(Lys25Glu) (red residue). Transmembrane domain 1 is represented in red and 2 in orange in the protein cartoon. The amino acid change is predicted to lead to misfolding of the protein and creation of another transmembrane domain.

After applying the suitable pipeline and filters, among the SNVs selected for hereditary spastic paraplegia, one novel missense heterozygous change was found in III-3 and in the father: *REEP1*: c.73A>G; p.(Lys25Glu) (NM_022912.2). The variant was confirmed by Sanger sequencing and was also found in the other brother and sister (Figure 4A), but a co-segregation study in other members of the paternal family was not possible due to their lack of collaboration. This variant was not reported previously in the literature, although it is known that heterozygous changes in this region of the *REEP1* gene cause spastic paraplegia, autosomal dominant type 31 (SPG31). All *in silico* predictors consulted for this variant predicted that it is pathogenic with a deleterious effect (MutationTaster^1^, PolyPhen-2^2^, SIFT^3^, PANTHER^4^, PROVEAN^5^, Mutation Assessor^6^). This prediction is based on the fact that the Lys25 is highly conserved and preserved over the course of the evolution and is a basic amino acid (aa) (pI=9.4), while Glu is an acid aa (pI=3.22), therefore, the variant might lead to drastic changes in the tertiary protein structure. Other *in silico* bioinformatic tools checked (The Protein Model Portal^7^; I-TASSER and QUARK^8^ and Protter^9^) predicted a conformational change in the protein that led to the passage of this residue from the lumen localization to the transmembrane domain (Figure 4B). However, if we applied the American College of Medical Genetics (ACMG) criteria for molecular variant classification using the InterVar software, the variant is classified as a VUS or with moderate criteria to be pathogenic/likely pathogenic (PM2, PP3, PP1). The reason for this assessment is that there are neither functional studies nor a co-segregation in more family members.

The duplication of the 15 chromosome (15q11.2–q13.1) was also confirmed by WES, in the brother studied in the *trio* (III-3) and his mother.

## Discussion

The relationship between an observed phenotype and its underlying genotype sometimes challenges the expected patterns of Mendelian inheritance. Deviations from these expectations lead us to the discovery of a more complicated genetic underground of disease. The present family, carrying an interstitial 15q duplication inherited from the mother and a *REEP1* variant inherited from the father, is an example of multiple or a possible dual molecular diagnosis, that is defined as involving more than one clinical diagnosis and more than one genetic locus, each segregating independently (12). This occurrence of dual molecular diagnosis in a single genome was reported in 3–7% of case series with a definitive diagnosis using whole exome sequencing (13). In a recent study of a large-scale clinical analysis of WES results, in which a molecular diagnosis was made in 28% of the cases, 4.9% of the patients received two or more molecular diagnoses, being variants of autosomal dominant diseases the most common pathogenic variants. In this line, the study also revealed that a combination of copy-number variants (CNVs) and single-nucleotide variants (SNVs) was found in 11.9% of those with multiple molecular diagnosis, so they only represent 0.57% of all the cases with a molecular diagnosis (13).

Chromosome 15q duplication syndrome (OMIM: 608636) is defined by the detection of one extra maternally derived copy of PWACR. Among all the genes located in this 15q11.2–q13.1 region, currently overexpression of the gene *UBE3A* is thought to be the principal pathological mechanism underlying these clinical features (14–16). Our findings reinforce this theory because methylation patterns and functional studies in the three siblings revealed an overexpression of *UBE3A*, whereas in the mother, the expression level was similar to the controls (Figure 3). These results also support that expressivity is variable even within families (15, 16): the older brother (III-3) associating refractory epilepsy with a moderate intellectual disability, compared to the milder cognitive phenotype of the young brother and to the sister, who presented with ADHD symptoms from school age and social anxiety from teens, but with normal IQ and no epilepsy. Surprisingly, although autistic behavior is one of the features frequently included to *UBE3A* copy number variants (9, 16, 17), none of the three siblings have this clinical finding. Interestingly, Piard et al. (10) reported a large family in which 12 individuals had a similar duplication that our family has—that is, between BP2 and BP3—and none of these 12 patients met the criteria for autism.

Regarding *ATP10A*, the expression was higher in both brothers than in the sister, suggesting a positive correlation of overexpression of *ATP10A* levels with clinical intensity (Figure 3). Interestingly, Hogart et al. (18) found that gender influenced allelic *ATP10A* expression, with the females as the ones that significantly express this gene less than the males. These results, together with the function of *ATP10A* as an aminophospholipid-transporting ATPase involved in cell signaling in the central nervous system (19), and the clinical findings in this family, suggest that *ATP10A* may play a role in cognitive deficit severity in the 15q duplication syndrome.

Since the 15q duplication found did not explain the HSP of the patients, deepening on the clinical history of the paternal family with some individuals with spastic paraparesis, an autosomal dominant inheritance was disclosed (Figure 1). A detailed neurological examination revealed corticospinal abnormal subclinical signs in the lower limbs of the father and also in the sister. WES studies were then performed and revealed a novel missense heterozygous change in *REEP1*: c.73A>G; p.(Lys25Glu) in the father and his three siblings (Figure 4A) that, according to *in silico* studies, is probably pathogenic, although the criteria of the ACMG considered it a VUS because there are neither functional studies nor co-segregation in more family members. *REEP1* variations are associated with SPG31 *pure* phenotype (OMIM: 610250), with autosomal dominant inheritance and a great heterogeneous clinical presentation, inter and intra families, with even unaffected carriers in some families (20). In some patients with SPG31, primarily upper motor neuron signs may associate peripheral neuropathy as in the patient III-3, or pes cavus without neuropathy as in the sister (III-4) and her father (II-5). No intellectual disability has been reported with *REEP1* variations so far.

## Concluding Remarks

In conclusion, our findings reinforce the association of maternally derived *UBE3A* overexpression with neurodevelopmental disorders, but the variability of phenotypes presents a challenge in the genetic counseling of the family. A second conclusion is that the genetic approach design in complex phenotypes is essential to get a definitive molecular diagnosis. In this family, a single genomic approach would have revealed only a part of the genotype and maybe underestimate the whole phenotype as an expanded phenotype instead of a dual diagnosis. For this reason, we suggest that a combination of CGH arrays and WES in complicated phenotypes is a useful tool as we have demonstrated here.

## Data Availability Statement

The sequencing data generated and analyzed in this study: *REEP1*: c.73A>G; p.[Lys25Glu (NM_022912.2)] can be found in the GenBank repository (https://www.ncbi.nlm.nih.gov/Genbank) with the accession number for this nucleotide sequence: BankIt2286781 REEP1_Ex2 MN734426.

## Ethics Statement

The study was approved by the Ethics committee for clinical research of Euskadi-Basque Country (CEIC-E: PI2014192). Written informed consent was obtained from all of the patients and relatives participating in the study, as well as for publication of the report.

## Author Contributions

SA-A and MI-Y conceived and designed the study. SA-A, AO-C, OB-C, MI-Y, MY-P, and IG provided blood samples, data acquisition, and clinical details of the patients. AH, NI, OV, GP, and AP performed the genetic analysis. SA-A, MT, AH, NI, OV, GP, and AP analyzed and interpreted the data. SA and MT wrote the manuscript. All authors reviewed and criticized it, approved the final version as submitted, and agreed to be accountable for all aspects of the work.

### Conflict of Interest

The authors declare that the research was conducted in the absence of any commercial or financial relationships that could be construed as a potential conflict of interest.

## Footnotes

^1^http://mutationtaster.org/

^2^http://genetics.bwh.harvard.edu/pph2/

^3^http://blocks.fhcrc.org/sift/SIFT.html

^4^http://panther.celera.com

^5^http://provean.jcvi.org/index.php

^6^http://mutationassessor.org/r3/

^7^www.proteinmodelportal.org

^8^https://zhanglab.ccmb.med.umich.edu

^9^http://wlab.ethz.ch/protter/

## Supplementary Material

Supplementary Figure 1 MS-MLPA results. Gene dosage analysis revealed that the three siblings and his mother presented a heterozygous duplication encompassing from at least the exon 1 of *MKNR3*, which starts at the genome position 23.810.454—GRCh37/hg19—to exon 3 of *OCA2* (whose end is at 28.277.211–GRCh37/hg19; duplicated probes in red, normal probes at 15p11 in green, and reference probes across the genome in gray). The three siblings show the same apparent hypermethylation pattern of *SNRPN:* alt-TSS-DMR; *SNURF*: TSS-DMR, and *MAGEL2*: TSS-DMR, opposite to the mother who shows apparent hypomethylation; MS-MLPA study was also performed in the father.
