# Supplementary material for: Hereditary Spastic Paraplegia and Intellectual Disability: Clinicogenetic Lessons From a Family Suggesting a Dual Genetics Diagnosis
Source: Front Neurol. 2020 Feb 14;11:41. doi: 10.3389/fneur.2020.00041 (PMC7033498; doi:10.3389/fneur.2020.00041)
Supplement: Supplementary Figure 1 — MS-MLPA results. Gene dosage analysis revealed that the three siblings and his mother presented a heterozygous duplication encompassing from at least the exon 1 of MKNR3, which starts at the genome position 23.810.454—GRCh37/hg19—to exon 3 of OCA2 (whose end is at 28.277.211–GRCh37/hg19; duplicated probes in red, normal probes at 15p11 in green, and reference probes across the genome in gray). The three siblings show the same apparent hypermethylation pattern of SNRPN: alt-TSS-DMR; SNURF: TSS-DMR, and MAGEL2: TSS-DMR, opposite to the mother who shows apparent hypomethylation; MS-MLPA study was also performed in the father. [file Presentation_1.PPTX]

## Slide 1
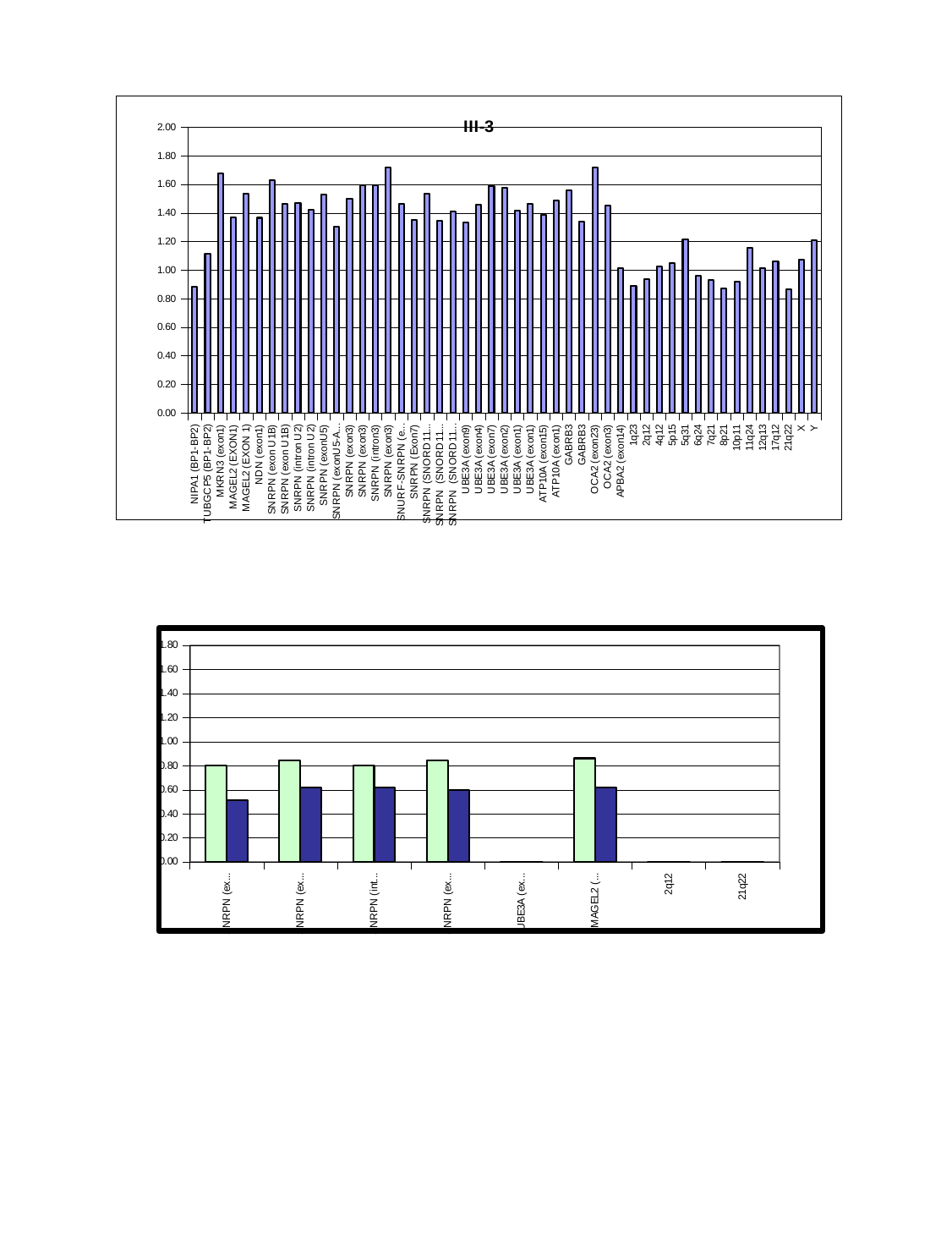

### Chart: III-3
| Category | 1158 |
|---|---|
| NIPA1 (BP1-BP2) | 0.8862816523673333 |
| TUBGCP5 (BP1-BP2) | 1.1166042045796096 |
| MKRN3 (exon1) | 1.6776413574409055 |
| MAGEL2 (EXON1) | 1.3698771319454541 |
| MAGEL2 (EXON 1) | 1.5371676660031277 |
| NDN (exon1) | 1.3664564825282168 |
| SNRPN (exon U1B) | 1.6308326179683885 |
| SNRPN (exon U1B) | 1.4642091444209635 |
| SNRPN (intron U2) | 1.4681176110380545 |
| SNRPN (intron U2) | 1.4245346111024608 |
| SNRPN (exonU5) | 1.5273237526884147 |
| SNRPN (exonU5-AS-SRO) | 1.304714557043682 |
| SNRPN (exon3) | 1.4982401527354194 |
| SNRPN (exon3) | 1.5965867343087528 |
| SNRPN (intron3) | 1.5922112108234585 |
| SNRPN (exon3) | 1.7187536653612718 |
| SNURF-SNRPN (exon3) | 1.465084430288227 |
| SNRPN (Exon7) | 1.3520846583069703 |
| SNRPN (SNORD116-1) | 1.532957731486972 |
| SNRPN (SNORD116-11) | 1.3477590437667015 |
| SNRPN (SNORD116-23) | 1.4097526419984747 |
| UBE3A (exon9) | 1.3331905605574257 |
| UBE3A (exon4) | 1.4607323483349648 |
| UBE3A (exon7) | 1.5901229633555773 |
| UBE3A (exon2) | 1.5749718458095023 |
| UBE3A (exon1) | 1.4159379058553185 |
| UBE3A (exon1) | 1.464399446554759 |
| ATP10A (exon15) | 1.3874500564898589 |
| ATP10A (exon1) | 1.4893565843466063 |
| GABRB3 | 1.5604808944313249 |
| GABRB3 | 1.34130741388811 |
| OCA2 (exon23) | 1.7194639872275614 |
| OCA2 (exon3) | 1.4539513639262625 |
| APBA2 (exon14) | 1.015641229950212 |
| 1q23 | 0.8895343190991224 |
| 2q12 | 0.9358009248241331 |
| 4q12 | 1.02719366267094 |
| 5p15 | 1.04691542388599 |
| 5q31 | 1.2161896747524032 |
| 6q24 | 0.9634639253757636 |
| 7q21 | 0.9313018507529685 |
| 8p21 | 0.8713283460038294 |
| 10p11 | 0.9199601365235477 |
| 11q24 | 1.1562301425794697 |
| 12q13 | 1.0135280694049993 |
| 17q12 | 1.064158685709979 |
| 21q22 | 0.8659884843417094 |
| X | 1.074095780917399 |
| Y | 1.2086623129473146 |
### Chart
| Category | 1158 | Control |
|---|---|---|
| SNRPN (exon3) | 0.8052456896579444 | 0.5136791012403441 |
| SNRPN (exon3) | 0.8431174899915732 | 0.6191518798622432 |
| SNRPN (intron3) | 0.799141541897942 | 0.6206126211857144 |
| SNRPN (exon3) | 0.8465917222799529 | 0.5974015453901058 |
| UBE3A (exon1) | 0.0 | 0.0 |
| MAGEL2 (EXON 1) | 0.8616487340900425 | 0.6164764296868456 |
| 2q12 | 0.0 | 0.0 |
| 21q22 | 0.0 | 0.0 |

## Slide 2
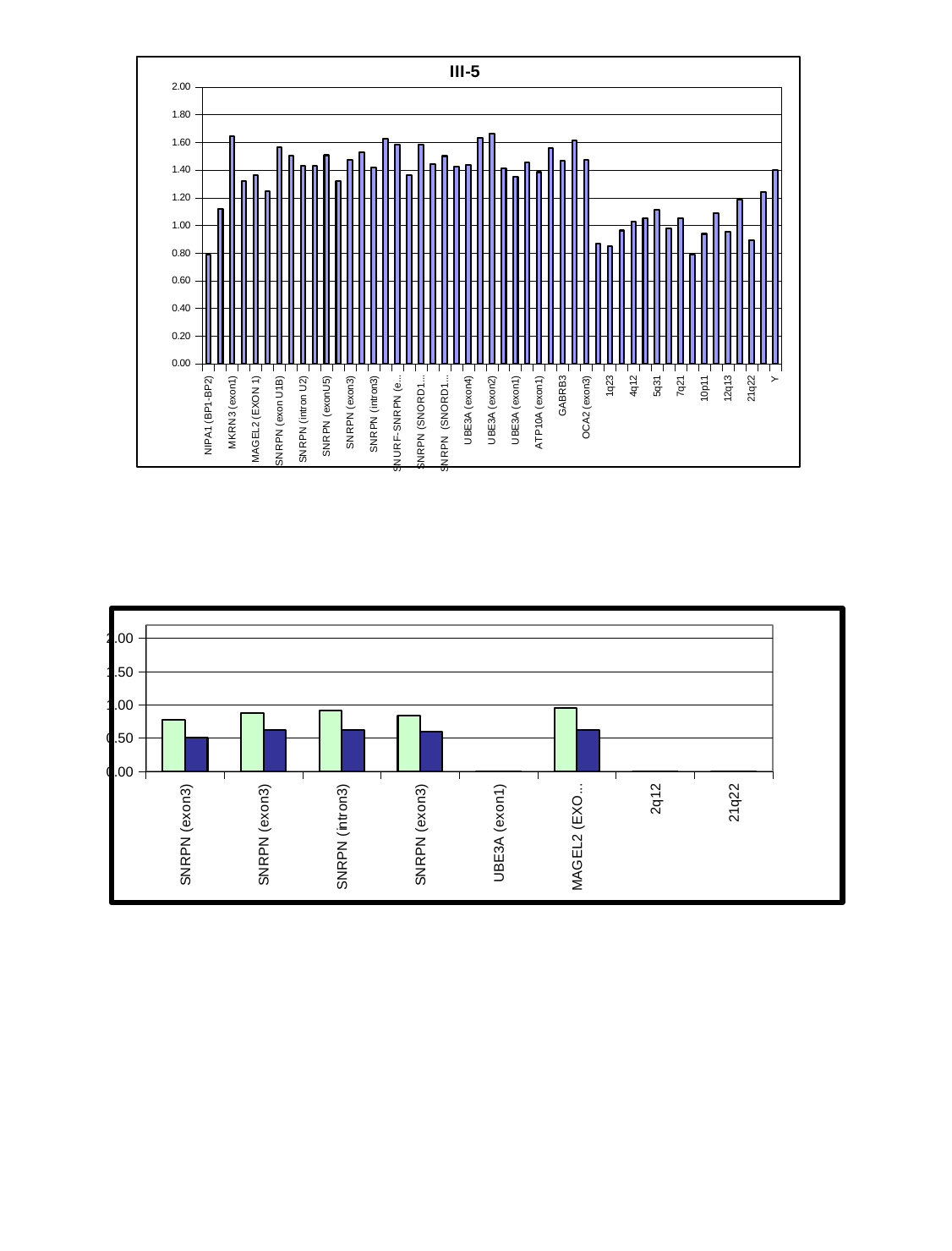

### Chart: III-5
| Category | 1159 |
|---|---|
| NIPA1 (BP1-BP2) | 0.7905954603106902 |
| TUBGCP5 (BP1-BP2) | 1.1216584638617626 |
| MKRN3 (exon1) | 1.6476559079256894 |
| MAGEL2 (EXON1) | 1.3220465925913256 |
| MAGEL2 (EXON 1) | 1.363143277914641 |
| NDN (exon1) | 1.2468694621006604 |
| SNRPN (exon U1B) | 1.5683365260721673 |
| SNRPN (exon U1B) | 1.5054976496711547 |
| SNRPN (intron U2) | 1.4335718302233733 |
| SNRPN (intron U2) | 1.4326131960610418 |
| SNRPN (exonU5) | 1.5073738884053998 |
| SNRPN (exonU5-AS-SRO) | 1.3227423281711697 |
| SNRPN (exon3) | 1.475294474034356 |
| SNRPN (exon3) | 1.530596731588713 |
| SNRPN (intron3) | 1.4174846397921024 |
| SNRPN (exon3) | 1.6239735293610842 |
| SNURF-SNRPN (exon3) | 1.5820102991631246 |
| SNRPN (Exon7) | 1.364116775376535 |
| SNRPN (SNORD116-1) | 1.5839202849686527 |
| SNRPN (SNORD116-11) | 1.4422989442520806 |
| SNRPN (SNORD116-23) | 1.501538614108766 |
| UBE3A (exon9) | 1.424076199782453 |
| UBE3A (exon4) | 1.4397003956662595 |
| UBE3A (exon7) | 1.6311701561735275 |
| UBE3A (exon2) | 1.6629897370843343 |
| UBE3A (exon1) | 1.4141228737473062 |
| UBE3A (exon1) | 1.35365749106782 |
| ATP10A (exon15) | 1.4541511776142935 |
| ATP10A (exon1) | 1.3851191706817714 |
| GABRB3 | 1.557324038676268 |
| GABRB3 | 1.4702374432015042 |
| OCA2 (exon23) | 1.614216100491865 |
| OCA2 (exon3) | 1.4713617480206636 |
| APBA2 (exon14) | 0.8697278019185816 |
| 1q23 | 0.8521316289853383 |
| 2q12 | 0.9635956377658067 |
| 4q12 | 1.0291120697901912 |
| 5p15 | 1.0494648360148504 |
| 5q31 | 1.110643393919063 |
| 6q24 | 0.9783630244371041 |
| 7q21 | 1.054973901298956 |
| 8p21 | 0.7885458634361903 |
| 10p11 | 0.9388731331728085 |
| 11q24 | 1.086927384113929 |
| 12q13 | 0.9554925632913325 |
| 17q12 | 1.185618577015188 |
| 21q22 | 0.8957563535614447 |
| X | 1.2441638157684345 |
| Y | 1.3980323785365276 |
### Chart
| Category | 1159 | Control |
|---|---|---|
| SNRPN (exon3) | 0.7764251602562952 | 0.5136791012403441 |
| SNRPN (exon3) | 0.8790456108739758 | 0.6191518798622432 |
| SNRPN (intron3) | 0.9158777388791768 | 0.6206126211857144 |
| SNRPN (exon3) | 0.8418492139924801 | 0.5974015453901058 |
| UBE3A (exon1) | 0.0 | 0.0 |
| MAGEL2 (EXON 1) | 0.9478866896138827 | 0.6164764296868456 |
| 2q12 | 0.0 | 0.0 |
| 21q22 | 0.0 | 0.0 |

## Slide 3
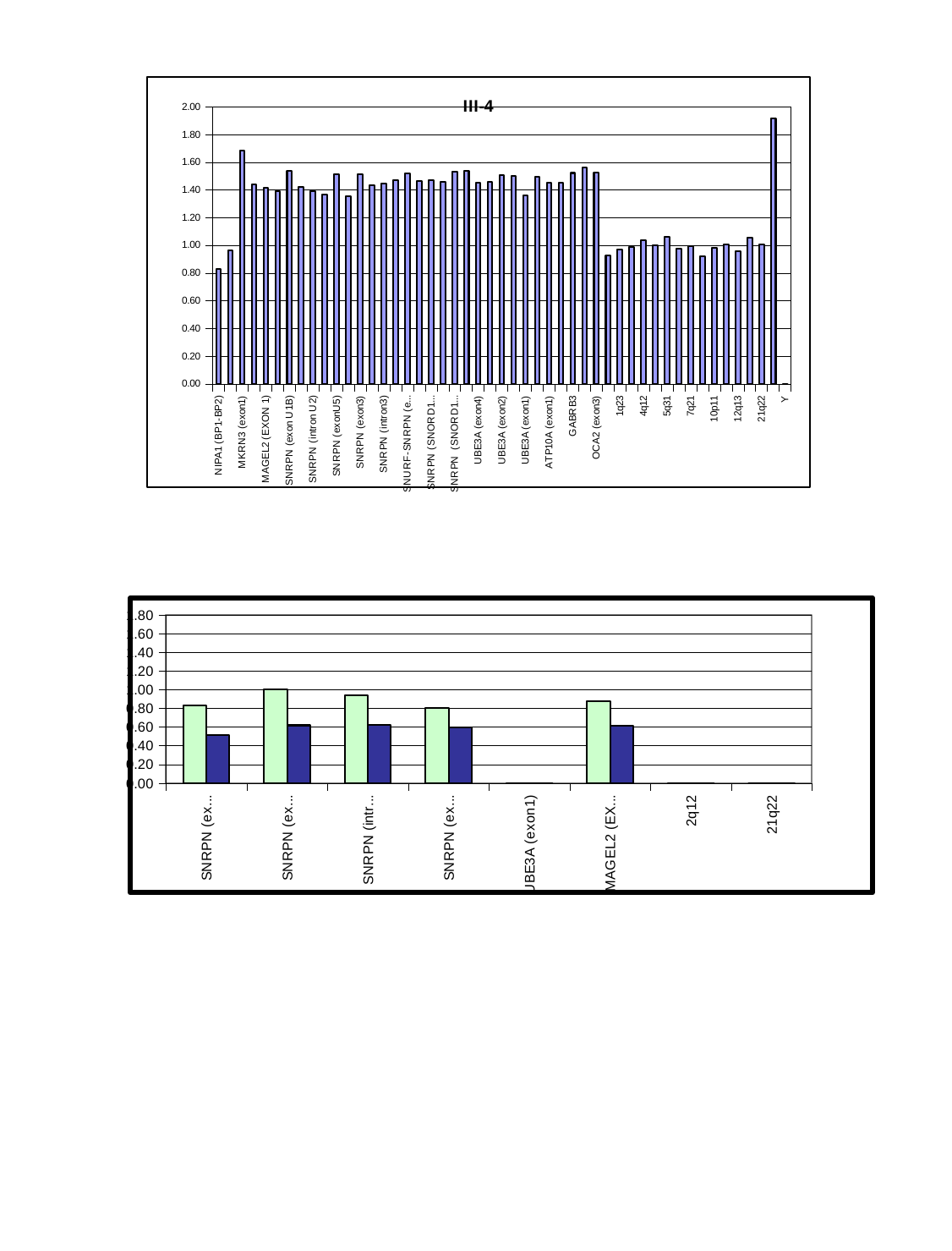

### Chart: III-4
| Category | 1158-HA |
|---|---|
| NIPA1 (BP1-BP2) | 0.8327881687828287 |
| TUBGCP5 (BP1-BP2) | 0.9634009973996086 |
| MKRN3 (exon1) | 1.687390058666337 |
| MAGEL2 (EXON1) | 1.4391457084166652 |
| MAGEL2 (EXON 1) | 1.4184680853890708 |
| NDN (exon1) | 1.389621030137128 |
| SNRPN (exon U1B) | 1.5384615365861702 |
| SNRPN (exon U1B) | 1.4253143911355322 |
| SNRPN (intron U2) | 1.3900254945482216 |
| SNRPN (intron U2) | 1.3691271634314424 |
| SNRPN (exonU5) | 1.5165843546395308 |
| SNRPN (exonU5-AS-SRO) | 1.3558431591403133 |
| SNRPN (exon3) | 1.5166691153428262 |
| SNRPN (exon3) | 1.432499713973467 |
| SNRPN (intron3) | 1.4443370184800262 |
| SNRPN (exon3) | 1.471305577224132 |
| SNURF-SNRPN (exon3) | 1.521520281927379 |
| SNRPN (Exon7) | 1.4656722015061625 |
| SNRPN (SNORD116-1) | 1.4713570760581531 |
| SNRPN (SNORD116-11) | 1.4593766744144134 |
| SNRPN (SNORD116-23) | 1.5312145222687839 |
| UBE3A (exon9) | 1.5358637888833235 |
| UBE3A (exon4) | 1.455062917636171 |
| UBE3A (exon7) | 1.4577761635830593 |
| UBE3A (exon2) | 1.5068904868047621 |
| UBE3A (exon1) | 1.502357170499879 |
| UBE3A (exon1) | 1.3611746356425065 |
| ATP10A (exon15) | 1.4934270188853322 |
| ATP10A (exon1) | 1.4551310618579383 |
| GABRB3 | 1.4504980654342434 |
| GABRB3 | 1.5234574398382632 |
| OCA2 (exon23) | 1.564828392258393 |
| OCA2 (exon3) | 1.5258381402574435 |
| APBA2 (exon14) | 0.926781929124567 |
| 1q23 | 0.9729048344529186 |
| 2q12 | 0.990555271227573 |
| 4q12 | 1.0373246940604863 |
| 5p15 | 0.9998436123087376 |
| 5q31 | 1.0627279642975391 |
| 6q24 | 0.9773395903355495 |
| 7q21 | 0.9939522310605545 |
| 8p21 | 0.9212846125243782 |
| 10p11 | 0.9805983596243342 |
| 11q24 | 1.0074414872030033 |
| 12q13 | 0.9553613046669476 |
| 17q12 | 1.0568729965950325 |
| 21q22 | 1.0045486777775443 |
| X | 1.9177272081626537 |
| Y | 0.0 |
### Chart
| Category | 1158-HA | Control |
|---|---|---|
| SNRPN (exon3) | 0.8334353019562589 | 0.5136791012403441 |
| SNRPN (exon3) | 1.0033223154320052 | 0.6191518798622432 |
| SNRPN (intron3) | 0.9414720926081745 | 0.6206126211857144 |
| SNRPN (exon3) | 0.8028174880881477 | 0.5974015453901058 |
| UBE3A (exon1) | 0.0 | 0.0 |
| MAGEL2 (EXON 1) | 0.8798599246921723 | 0.6164764296868456 |
| 2q12 | 0.0 | 0.0 |
| 21q22 | 0.0 | 0.0 |

## Slide 4
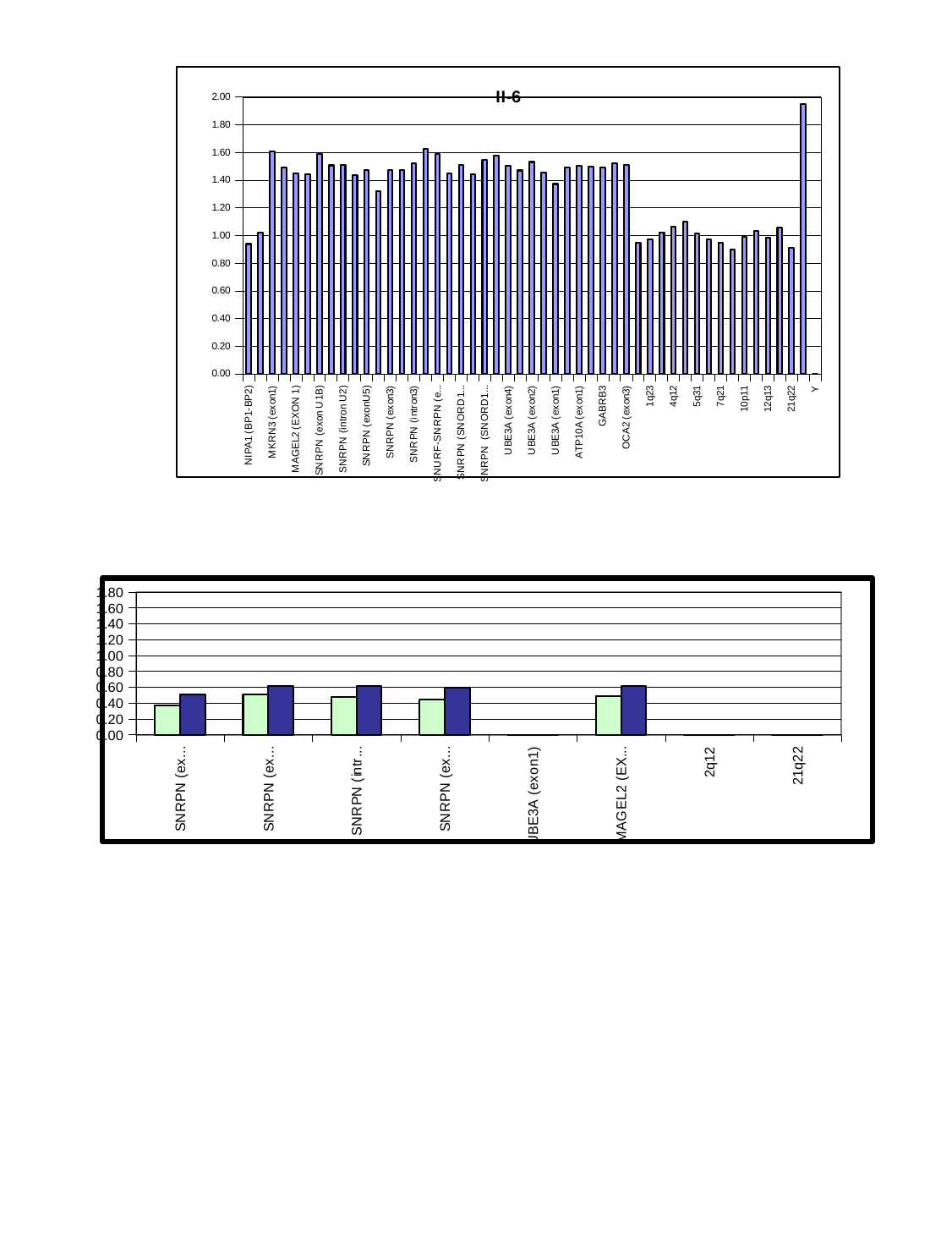

### Chart: II-6
| Category | 1160 |
|---|---|
| NIPA1 (BP1-BP2) | 0.9378731642764576 |
| TUBGCP5 (BP1-BP2) | 1.0230295032916186 |
| MKRN3 (exon1) | 1.6058945338431945 |
| MAGEL2 (EXON1) | 1.4911253612564266 |
| MAGEL2 (EXON 1) | 1.4468698469041341 |
| NDN (exon1) | 1.4431050316058518 |
| SNRPN (exon U1B) | 1.5862928530405516 |
| SNRPN (exon U1B) | 1.5064856827508213 |
| SNRPN (intron U2) | 1.5083414394524128 |
| SNRPN (intron U2) | 1.4383920153946532 |
| SNRPN (exonU5) | 1.472488046440855 |
| SNRPN (exonU5-AS-SRO) | 1.3182729919788743 |
| SNRPN (exon3) | 1.4746750839060776 |
| SNRPN (exon3) | 1.4702286917520373 |
| SNRPN (intron3) | 1.518712881074167 |
| SNRPN (exon3) | 1.626928276770548 |
| SNURF-SNRPN (exon3) | 1.58814361157258 |
| SNRPN (Exon7) | 1.4476456473577966 |
| SNRPN (SNORD116-1) | 1.510492502307116 |
| SNRPN (SNORD116-11) | 1.44200990344901 |
| SNRPN (SNORD116-23) | 1.544571763259834 |
| UBE3A (exon9) | 1.5769428375888759 |
| UBE3A (exon4) | 1.5029463847709026 |
| UBE3A (exon7) | 1.4697094494308542 |
| UBE3A (exon2) | 1.5304463588425525 |
| UBE3A (exon1) | 1.4532777887523312 |
| UBE3A (exon1) | 1.3718247466032474 |
| ATP10A (exon15) | 1.4934885383051193 |
| ATP10A (exon1) | 1.504668891491606 |
| GABRB3 | 1.500127054610292 |
| GABRB3 | 1.4890076041448062 |
| OCA2 (exon23) | 1.5224186410248042 |
| OCA2 (exon3) | 1.510768670766824 |
| APBA2 (exon14) | 0.9481785026100938 |
| 1q23 | 0.9685413459667933 |
| 2q12 | 1.0191586168198565 |
| 4q12 | 1.0632713093691308 |
| 5p15 | 1.0976193656701134 |
| 5q31 | 1.0159611532327066 |
| 6q24 | 0.9733426372910117 |
| 7q21 | 0.9456712265007335 |
| 8p21 | 0.8988826868940881 |
| 10p11 | 0.9910787242542184 |
| 11q24 | 1.0321502351168497 |
| 12q13 | 0.9838288961337399 |
| 17q12 | 1.0546232752873164 |
| 21q22 | 0.911109062820621 |
| X | 1.9520660787833162 |
| Y | 0.0 |
### Chart
| Category | 1160 | Control |
|---|---|---|
| SNRPN (exon3) | 0.37289760871752103 | 0.5136791012403441 |
| SNRPN (exon3) | 0.5040591656662214 | 0.6191518798622432 |
| SNRPN (intron3) | 0.4730951363247651 | 0.6206126211857144 |
| SNRPN (exon3) | 0.4471412981318604 | 0.5974015453901058 |
| UBE3A (exon1) | 0.0 | 0.0 |
| MAGEL2 (EXON 1) | 0.4829306125674743 | 0.6164764296868456 |
| 2q12 | 0.0 | 0.0 |
| 21q22 | 0.0 | 0.0 |

## Slide 5
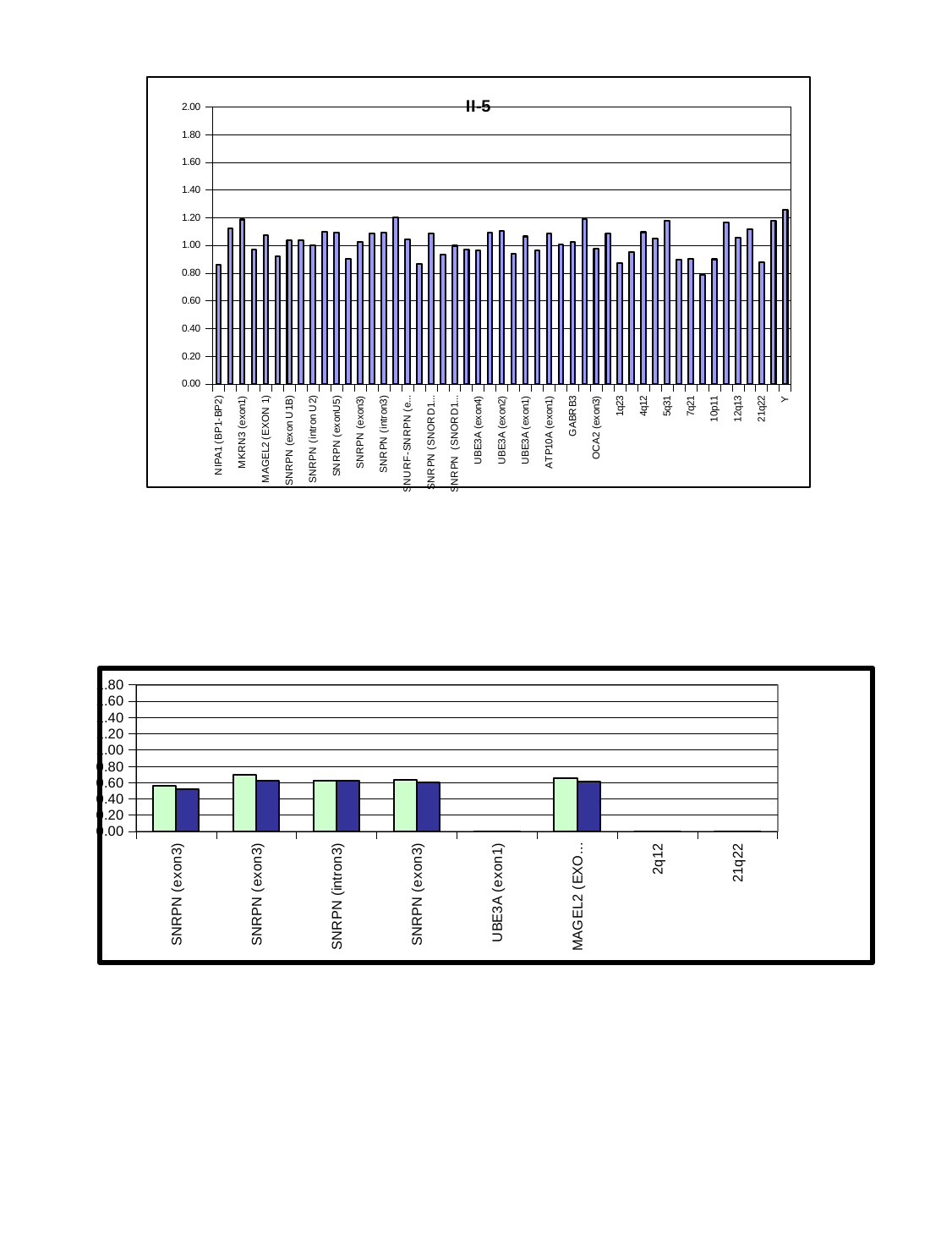

### Chart: II-5
| Category | 1161 |
|---|---|
| NIPA1 (BP1-BP2) | 0.8585211173014653 |
| TUBGCP5 (BP1-BP2) | 1.122283588427362 |
| MKRN3 (exon1) | 1.1873474750004083 |
| MAGEL2 (EXON1) | 0.9724510101197084 |
| MAGEL2 (EXON 1) | 1.0753314089639823 |
| NDN (exon1) | 0.9227356651795325 |
| SNRPN (exon U1B) | 1.0380896396650416 |
| SNRPN (exon U1B) | 1.039024909516079 |
| SNRPN (intron U2) | 0.9999943822129672 |
| SNRPN (intron U2) | 1.096686632158544 |
| SNRPN (exonU5) | 1.0934696353533453 |
| SNRPN (exonU5-AS-SRO) | 0.9021849086819045 |
| SNRPN (exon3) | 1.02524908410089 |
| SNRPN (exon3) | 1.0865304498638628 |
| SNRPN (intron3) | 1.0938552728741187 |
| SNRPN (exon3) | 1.2032272813565026 |
| SNURF-SNRPN (exon3) | 1.0464264774675227 |
| SNRPN (Exon7) | 0.865536100299956 |
| SNRPN (SNORD116-1) | 1.0845346015871067 |
| SNRPN (SNORD116-11) | 0.9332563689940289 |
| SNRPN (SNORD116-23) | 0.9981329017420558 |
| UBE3A (exon9) | 0.9680327829018301 |
| UBE3A (exon4) | 0.9664570785027258 |
| UBE3A (exon7) | 1.090317393157206 |
| UBE3A (exon2) | 1.103788783127024 |
| UBE3A (exon1) | 0.9408357938822318 |
| UBE3A (exon1) | 1.0648476691107303 |
| ATP10A (exon15) | 0.9647869315618135 |
| ATP10A (exon1) | 1.0886980901331473 |
| GABRB3 | 1.0058180565167227 |
| GABRB3 | 1.0269530281382453 |
| OCA2 (exon23) | 1.1886182026078238 |
| OCA2 (exon3) | 0.9767568992881211 |
| APBA2 (exon14) | 1.0861940200015792 |
| 1q23 | 0.8698624116545596 |
| 2q12 | 0.9532177136694205 |
| 4q12 | 1.0959317367603394 |
| 5p15 | 1.0501126829113379 |
| 5q31 | 1.1791854545622555 |
| 6q24 | 0.8993983244270399 |
| 7q21 | 0.9006729392623537 |
| 8p21 | 0.7897360515267554 |
| 10p11 | 0.9000472525085249 |
| 11q24 | 1.1681546894673445 |
| 12q13 | 1.0559897765259179 |
| 17q12 | 1.1167163272533596 |
| 21q22 | 0.8772357557543856 |
| X | 1.1806323464078474 |
| Y | 1.2549257743585505 |
### Chart
| Category | 1161 | Control |
|---|---|---|
| SNRPN (exon3) | 0.5628661511404028 | 0.5136791012403441 |
| SNRPN (exon3) | 0.6963080689066722 | 0.6191518798622432 |
| SNRPN (intron3) | 0.6262767416299844 | 0.6206126211857144 |
| SNRPN (exon3) | 0.6307498709421506 | 0.5974015453901058 |
| UBE3A (exon1) | 0.0 | 0.0 |
| MAGEL2 (EXON 1) | 0.6553691347443801 | 0.6164764296868456 |
| 2q12 | 0.0 | 0.0 |
| 21q22 | 0.0 | 0.0 |
